# Deoxynivalenol and Fumonisin B1 in Gilthead Seabream Diets: Impact on Growth Performance, Hematology, Immunology, and Histopathology

**DOI:** 10.3390/biom16091258

**Published:** 2026-08-31

**Authors:** Christina Papadouli, Sofia Vardali, Theodoros Karatzinos, Fotis Lykotrafitis, Myrto Maniaki, Panagiota Panagiotaki, George Rigos, Ioannis Nengas, Morgane Henry, Chrysanthi Nikoloudaki, Dimitra Kogiannou, Petros Chronopoulos, Eleni Golomazou

**Affiliations:** 1 Department of Ichthyology and Aquatic Environment-Aquaculture Laboratory, School of Agricultural Sciences, University of Thessaly, Fytokou Str., 38446 Volos, Greeceflikotrafitis@patt.gov.gr (F.L.);; 2 Institute of Marine Biology, Biotechnology, and Aquaculture, Hellenic Centre for Marine Research, 46.7 km Athens-Sounion, 19013 Attiki, Greece; grigos@hcmr.gr (G.R.); morgane@hcmr.gr (M.H.); cnikol@hcmr.gr (C.N.); dkogiannou@hcmr.gr (D.K.);

**Keywords:** mycotoxins, *Sparus aurata*, aquafeeds, health protection, welfare, genotoxicity

## Abstract

As aquafeed formulations include more plant-based materials, mycotoxin contamination is becoming relevant for fish farming. Deoxynivalenol (DON) and fumonisin B1 (FB1) frequently occur in fish feeds and may impair fish performance and health. This study evaluated the impact of DON and FB1 on growth and health parameters in gilthead seabream (*Sparus aurata*). Fish were fed *ad libitum* for seven weeks with six diets assessed in triplicate: DON A (300 ppb), DON B (2000 ppb), DON C (5000 ppb), FB1 A (5 ppb), FB1 B (10 ppb), FB1 C (40 ppb). The highest contamination levels significantly reduced feed intake, body weight, total length, and biomass, while body weight was also reduced in the lowest FB1 treatment. The feed conversion ratio increased in exposed fish, whereas the specific growth rate decreased in DON B, DON C, FB1 A, and FB1 C. Both mycotoxins altered hematological profiles, immune-related enzymatic activities, and liver morphology. DNA damage increased with exposure level and was greatest at the highest DON concentration. Overall, this study shows that short-term dietary exposure can impair seabream performance and health, supporting stricter mycotoxin monitoring in fish feeds and research on chronic exposure, recovery, and species-specific safety thresholds.

## 1. Introduction

In recent decades, aquaculture has expanded rapidly, with global production increasing by 204% between 2000 and 2022 and a mean annual growth rate of 5.2% [1]. This growth has historically depended on aquafeeds rich in marine-derived ingredients, particularly fishmeal and fish oil, which provide key nutrients for farmed aquatic species. However, the availability and sustainability of these resources are increasingly challenged, as the proportion of fish stocks within biologically sustainable levels declined between 2019 and 2021, while unsustainably exploited stocks have been increasing steadily since the mid-1970s [1]. Aquaculture is estimated to consume approximately 69% of global fishmeal and 75% of global fish oil production [2], and the rising demand for these ingredients has contributed to pressure on pelagic fish stocks and to increased market prices [3,4]. Consequently, the sector is progressively shifting towards alternative protein sources, including plant ingredients, to support sustainable production without compromising product quality [5]. Nevertheless, greater reliance on plant-derived materials may introduce anti-nutritional factors and feed contaminants. Among these, mycotoxins are of particular concern, as they are frequently associated with plant-based feedstuffs and may affect both aquafeed safety and fish health [6,7,8].

Mycotoxins are toxic compounds derived from several fungal species and are regularly reported in agricultural commodities intended for feed production [9]. Their presence in feedstuffs is a concern for farmed animals and consumers, since exposure may occur directly through contaminated crops and crop-derived products or indirectly via products originating from animals exposed to contaminated diets [10]. Consequently, when fish ingest a combination of mycotoxins, it may not only affect the animal’s well-being but also potentially cross the food chain to the consumers and cause significant health issues. Numerous mycotoxins have been identified, with those of primary importance in feed being predominantly synthesized by fungi from the genera *Aspergillus*, *Fusarium*, *Penicillium*, *Claviceps*, and *Alternaria* [11]. Fumonisin B1 [FB1] and deoxynivalenol [DON] are among the mycotoxins most often identified in aquafeeds, frequently at considerable concentrations [7]. These mycotoxins have the potential to cause adverse effects on farmed fish, resulting in significant financial impacts linked to impaired production efficiency, increased sensitivity to infections and mortality [12].

Survey data from European aquafeeds indicate that DON is among the most commonly detected mycotoxins in both finished fish feeds and their ingredients [13]. It is primarily produced by *Fusarium* fungi that infect crops prior to harvest, making prevention strategies particularly challenging [14]. DON shows considerable stability during heat treatment; therefore, the high temperatures reached during feed processing cannot eliminate DON or ensure its absence from the final fish feed product [15]. Previous studies on various fish species, including rainbow trout (*Oncorhynchus mykiss*) [15], Nile tilapia (*Oreochromis niloticus*) [16], common carp (*Cyprinus carpio*) [17], and Atlantic salmon (*Salmo salar*) [18], have demonstrated the negative effects of DON-contaminated diets on weight gain, nutrient utilization, immune parameters, oxidative stress, and organ damage.

Fumonisins are produced by several *Fusarium* species and share similar polar chemical structures. Among them, FB1 is regarded as the most toxic form, with maize and maize-derived products representing its main contaminated substrates before harvest [19]. These toxins are associated with various health issues, including liver and kidney damage, as well as disruptions in sphingolipid metabolism, particularly in pigs, ruminants, rabbits, poultry, and some fish species [20]. Elucidating the prevalence and impact of fumonisins in fish feed is crucial to mitigating their adverse effects on fish health and their subsequent implications for human consumption. Fumonisins in fish feed can cause organ damage, immunosuppression, delayed growth, and metabolic alterations, potentially resulting in mortality [21]. Consistent with these reported effects, experimental studies in Nile tilapia (*Oreochromis niloticus*), African catfish (*Clarias gariepinus*), and juvenile grass carp (*Ctenopharyngodon idella*) have shown that dietary FB1 exposure can impair growth performance, feed utilization, hematological and biochemical parameters, and intestinal health [22,23,24].

Mediterranean marine aquaculture is largely centered on the sea-cage farming of carnivorous fish. The sector is dominated by gilthead seabream (*Sparus aurata*) and European sea bass (*Dicentrarchus labrax*), which represent 45.8% and 44.6% of total Mediterranean marine production, respectively [25]. In Greece, marine fish farming of these two dominant species occupies a dominant position, with an estimated annual production of 121,300 tons representing about 92% of the total Greek aquaculture value [26]. Despite the dynamics of Mediterranean aquaculture, studies on mycotoxins affecting cultured Mediterranean fish remain limited, although the occurrence of mycotoxins in aquaculture feeds in the Mediterranean region has been established [27], particularly considering the current era of climate change.

In this study, gilthead seabreams were fed diets containing different levels of DON or FB1 to assess their effects on growth performance and selected health parameters. The selected health endpoints included hematological parameters, innate immune responses, hepatic DNA damage, and liver histopathology. Given the relevance of this species for Mediterranean aquaculture, the results may provide useful information on its response to dietary mycotoxin exposure.

## 2. Materials and Methods

### 2.1. Ethical Approval

All experimental procedures involving fish adhered to EU Directive 2010/63/EU concerning the protection of animals used for scientific purposes. The personnel responsible for the experimental protocol, animal care, procedures, sampling, and euthanasia were trained and operated under the supervision of FELASA-accredited scientists (Functions A–D). The experimental protocol was approved by the University Ethical Committee (Approval No 18403/05-09-2019). The trial was conducted at the registered experimental aquarium facilities of the Aquaculture Laboratory (EL-43BIO/exp-01), Department of Ichthyology and Aquatic Environment (DIAE), University of Thessaly (Volos, Greece).

### 2.2. Experimental Fish

Gilthead seabreams were procured from a commercial aquaculture facility (Fish Farming Facility: GR06FISH0008, Fthiotida, Greece) and maintained within the recirculating system laboratory facilities at the University of Thessaly (Thessaly, Volos, Greece). A total of 630 fish, with an average weight of 3.40 ± 0.01 g and an average length of 6.40 ± 0.01 cm, were randomly allocated to 21 glass aquaria (30 fish per aquarium in triplicate groups), each with a capacity of 125 L (50 × 50 × 50 cm), forming part of the laboratory recirculating aquaculture system The fish were acclimated for 10 days to experimental conditions prior to the commencement of the study. During this period, they were provided with commercial fish feed once daily. Throughout the acclimation period, water physicochemical parameters were regularly monitored and maintained at 21 ± 1 °C for temperature, pH 8.0 ± 0.4, salinity 33 ± 0.5 g/L, total ammonia nitrogen below 0.1 mg/L, and dissolved oxygen at approximately 8 mg/L, under a 12 h light:12 h dark photoperiod. These water quality parameters and photoperiod conditions were identical to those maintained throughout the subsequent experimental feeding period.

### 2.3. Experimental Diet Formulation

The mycotoxins used in this study were supplied by Cayman Chemical (Ann Arbor, MI, USA). DON (Item No. 11428) and FB1 (Item No. 62580) were incorporated into diets at various contamination levels. Six experimental diets were formulated, each containing different concentrations of mycotoxins DON A (300 ppb), DON B (2000 ppb), DON C (5000 ppb), FB1 A (5 ppb), FB1 B (10 ppb), and FB1 C (40 ppb). The experimental concentrations were selected to encompass low, intermediate, and high exposure levels, with DON concentrations of 300, 2000, and 5000 ppb reflecting commercially plausible contamination, previously reported effects in fish, and the current EU guidance value, respectively [28,29], while FB1 concentrations of 5, 10, and 40 ppb were based on preliminary unpublished evidence of hepatocellular alterations at 10 ppb and a dose-ranging approach to evaluate effects across increasing exposure levels. The control diet (CTRL) was entirely devoid of mycotoxins.

All experimental diets were formulated and produced in the facilities of the Hellenic Center for Marine Research (HCMR) to resemble typical commercial fish diets, incorporating fishmeal, fish oil, and plant protein sources, such as soybean and wheat meal, which were intentionally contaminated with mycotoxins (Table 1). Prior to incorporation into the experimental diets, DON and FB1 were reconstituted in ethanol at concentrations of 5 mg/mL and 1 mg/mL, respectively, according to the manufacturer’s instructions. The resulting mycotoxin solutions were then added to 10 mL of salmon oil, and the ethanol was allowed to evaporate in a fume hood for 72 h. Appropriate volumes of the resulting mycotoxin–oil mixtures were subsequently diluted in 200 mL of salmon oil before incorporation into the ingredient mixture used for pellet production, in order to promote homogeneous distribution of the mycotoxins throughout the experimental diets. All ingredients were weighed, ground, and mixed at the HCMR experimental fish feed production facility, and feed pellets were manufactured using cold pressing with a CLEXTRAL laboratory pellet extruder, followed by oil addition via vacuum coating (DINISSEN). The final diets were subsequently dried at 60 °C. The estimated feed composition was identical across all treatments, comprising 45.60% protein, 16.10% fat, 9.59% ash, 0.10% fiber, 20.60% nitrogen-free extract (NFE), 17.00% starch, 1.37% calcium, and 1.32% phosphorus. The experimental diets were stored at 4 °C during the experimental period.

The commercial forms of DON and FB1 were both processed and incorporated into the experimental diets at specified concentrations. To ensure homogenous distribution, the required quantities of mycotoxins were diluted in 200 mL of salmon fish oil before integration into the ingredient mixture to produce pellets. The experimental diets were stored at 4 °C during the experimental period.

### 2.4. Experimental Procedure

During the feeding trial, fish were offered the experimental diets by hand to apparent satiation twice per day, at 09:00 and 18:30, six days per week, for seven weeks. Feed intake and mortality were recorded daily. Fish behavior and general condition were also checked each day, including signs of abnormal behavior, such as irregular swimming, lethargy, hyperactivity, cannibalism, external lesions, or disease symptoms. Water quality was monitored throughout the trial and remained within the appropriate range for gilthead seabream, with the pH at 8.00 ± 0.40, ammonia (NH_3_/N) below 0.10 mg/L, salinity at 33.00 ± 0.50 g/L, and dissolved oxygen at 8 mg/L. Water temperature was kept at 21.00 ± 1.00 °C, under a 12 h light:12 h dark photoperiod. The recirculating aquaculture system was equipped with mechanical filtration using glass-wool filter media to retain solid particles, including uneaten feed and fecal material, and biological filtration based on nitrifying bacteria to support the removal of nitrogenous waste. Both mechanical and biological filters were inspected and maintained daily. In addition, the aquaria were siphoned daily to remove settled and suspended particulate matter and to prevent water turbidity. At the end of the experiment, fish were fasted for 24 h before sampling. Blood collection was performed under anaesthesia with tricaine methanesulfonate (MS-222) at a concentration of 15 mg/L (Sigma Aldrich, St. Louis, MO, USA), with five fish anaesthetized at a time for approximately 1–2 min. The fish were subsequently euthanized with an overdose of tricaine methanesulfonate (MS-222), following Directive 2010/63/EU and FELASA guidelines.

### 2.5. Growth Performance

The total wet weight and length of the fish were measured before euthanasia to calculate the growth parameters for each treatment using the following formulas:

Weight gain (WG, g) = final body weight (g) − initial body weight (g)(1)

Specific growth rate (SGR, %/day) = 100 × [ln (final body weight) − ln (initial body weight)]/days(2)

Feed conversion ratio (FCR) = feed intake (g)/wet weight gain (g) (3)

Survival (%) = 100 × number of fish at the end of the experiment/number of fish stocked(4)

### 2.6. Hematological Parameters

Blood samples were obtained from the caudal vein of 5 fish per aquarium, amounting to 24 fish per dietary group, using heparinized syringes. Fresh heparinized whole blood was used for the hematological analyses. Hematocrit (HCT) was determined using 75 mm microhematocrit capillary tubes, which were filled with blood to approximately 60–70% of their length, avoiding the formation of air bubbles, and sealed at one end. The samples were immediately centrifuged in a microhematocrit centrifuge at a fixed speed of 12,000 rpm for 10 min. The concentration of hemoglobin (g/dL) was assessed as previously described [30].

Erythrocyte (RBC) and leukocyte (WBC) counts were performed immediately after blood collection. Briefly, 20 μL of blood were mixed with 4 mL of 0.85% NaCl solution prepared in deionized water, resulting in a 1:200 dilution. The suspension was gently mixed to ensure homogeneous cell distribution, and 10 μL was loaded into a Neubauer hemocytometer (Hausser Scientific, Horsham, PA, USA). RBCs and WBCs were counted manually under a light microscope using the appropriate counting areas of the hemocytometer, and cell concentrations were calculated according to the dilution factor and chamber volume.

### 2.7. Immunological Parameters

Eight fish were anesthetized and sampled from each aquarium (24 fish/diet). Blood was collected from the caudal vein using syringes without heparin. The unheparinized blood was allowed to clot for 3 h at 4 °C and centrifuged at 10,000× *g* for 10 min, and the collected sera were frozen at −80 °C until immunological analyses were conducted. The antibacterial activity of the complement [31], the myeloperoxidase activity [32,33], the anti-protease and the ceruloplasmin [34], and the alkaline phosphatase [35] activities were assessed according to previously described established protocols without modifications. All parameters were evaluated using a microplate spectrophotometer (GeniosPro, Tecan, Austria) and data were collected and processed using Magellan 4.0. software.

### 2.8. Alkaline Single-Cell Gel Electrophoresis (SCGE)/Comet Assay

Liver tissue was collected, and the comet assay (alkaline single-cell gel electrophoresis) method was implemented for the detection of DNA damage [36]. The comet assay is a gel electrophoresis method used to measure DNA strand fragments in single cells using microscopy. In brief, liver tissues were removed, perfused in HBSS, minced and injected with collagenase (e.g., 10 IU). Then, the tissues were transferred to tubes, homogenized and centrifuged 3 times (4 °C, 3000 rpm, 5 min). Then, 40 μL of each sample’s suspension and 160 μL of low agarose were mixed in a tube, and 100 μL of this solution was embedded on agarose pre-coated slides and gently covered with coverslips. When the samples started to dry, the coverslips were removed and the slides were immersed in a lysis solution buffer (29.22 g NaCl, 7.45 g EDTA, 0.242 g Tris, 10% DMSO, 1% Triton X-100 and pH 10) overnight in the refrigerator (4 °C). Then, the slides were washed with deionized water and placed in a horizontal gel electrophoresis unit, immersed in 1.5 L electrophoresis buffer (4.5 g Sodium Hydroxide, 0.5583 EDTA, and deionized water, pH > 12) and left there for 20 min in sterile conditions. Then, electrophoresis was performed, where the samples were exposed to an electric field (30 V, 300 mA) for 15 min to orient negatively charged DNA fragments. After electrophoresis, the slides were neutralized gently with Tris buffer at pH 7.5 and dried overnight at room temperature. Finally, the slides were stained using fluorescent dye (Midori Green Advance DNA Stain, MG04, NIPPON Genetics EUROPE, Düren, Germany), and DNA was observed using a fluorescence microscope at 40× magnification, with an excitation filter range of 510–590 nm. The Comet Assay IVTM comet scoring system was used to evaluate DNA migration. Having undergone this process, broken DNA migrated out of the nucleus in the electric field, and the damaged cells resembled “comets” with a brightly fluorescent head (nucleus) and a “tail” (fragmented DNA). Tail intensity was used to analyze the integrity of DNA molecules in nuclei, thereby indicating the severity of DNA damage. Different tail intensities indicated diverse levels of damage-stress.

### 2.9. Histopathological Examination

Formalin-fixed tissues were processed and embedded in paraffin 24 h after sampling. For histological analysis, liver samples were obtained from three fish per treatment group and fixed in a 10% formaldehyde solution for a minimum of 24 h [37]. Subsequently, the samples were dehydrated using a Leica TP1020 tissue processor (Leica Biosystems Nussloch GmbH, Nussloch, Germany) using a graded series of ethanol and methanol, immersed in chloroform, and embedded in paraffin. Paraffin blocks were sectioned at a thickness of 3 μm using a Leica RM 2255 rotary microtome (Nussloch, Germany). The sections were de-paraffinized in xylene, rehydrated in a series of graded alcohol baths, and stained with hematoxylin and eosin (HE). The histopathological examination was performed using an Olympus VANOX-T light microscope (Olympus Optical Co., Tokyo, Japan) equipped with a Lumenera Infinity digital camera (Lumenera Corporation, Ottawa, ON, Canada). Digital images were captured at a resolution of 300 dpi.

### 2.10. Statistical Methods

All treatments were performed in triplicate, and the results are expressed as means ± standard deviation (SD), unless otherwise stated. For selected results presented in figures, values are expressed as means ± standard error of the mean (SEM), as indicated in the corresponding figure legends. Statistical analysis was performed using IBM SPSS Statistics version 26 (IBM Corp., Armonk, NY, USA). Data concerning growth performance, hematological, and immunological parameters were tested for normality and homogeneity of variances using Shapiro–Wilk and Levene’s tests, respectively, and they were transformed when required before being subjected to one-way analysis of variance (ANOVA) to determine statistically significant differences among treatments. ANOVA was followed by Tukey’s post hoc test. When normal distribution or homogeneity of variances was not achieved, non-parametric tests were performed, i.e., the Kruskal–Wallis test followed by the Games–Howell post hoc test. Differences were considered statistically significant at *p* < 0.05.

## 3. Results

### 3.1. Growth Performance

All growth performance parameters are given in Table 2. Feed consumption was influenced by mycotoxin concentration in a dose-dependent manner. Specifically, fish exposed to the highest levels of both DON (DON C group) and FB1 (FB1 C group) exhibited a reduction in feed intake per individual compared with the CTRL group. Consideration of total feed consumption further highlighted this pattern, with the DON C group showing a statistically significant decrease among all treatments. In addition to reduced feed intake, mycotoxin exposure had a pronounced impact on growth performance indicators. The total biomass and weight gain were significantly lower in most groups receiving mycotoxins than in the CTRL group, indicating the negative influence of both DON and FB1 on growth. The DON C group demonstrated the most severe suppression of growth parameters, presenting significantly lower biomass and weight gain than both the CTRL and other groups exposed to mycotoxins, highlighting the harmful effects of higher DON concentrations on growth efficiency.

### 3.2. Hematological Factors

Exposure to both mycotoxins affected the examined hematological parameters (Table 3). Hematocrit (HCT) values were numerically lower in all mycotoxin-treated groups than in the CTRL group. However, the reduction reached statistical significance only in the FB1 A group, whereas the remaining treatments showed intermediate values that did not differ significantly from either the CTRL or the FB1 A group. A significant decrease in hemoglobin levels was observed in the intermediate DON B group compared to the CTRL, while no significant differences from the CTRL were detected for the remaining treatments. Significant differences were also detected in red blood cell (RBC) counts. All groups exposed to DON exhibited higher RBC values compared to the CTRL, with the highest value shown in the DON C group. In contrast, the FB1 A group did not differ from the CTRL, while FB1 B showed an intermediate increase in RBC counts that was not statistically significant compared with the CTRL. The FB1 C group exhibited higher RBC counts. WBC counts were significantly higher in the DON B and DON C groups compared to the CTRL group, while the DON A and all FB1-treated groups did not differ significantly from the CTRL.

### 3.3. Immunological Factors

**Figure 1:**
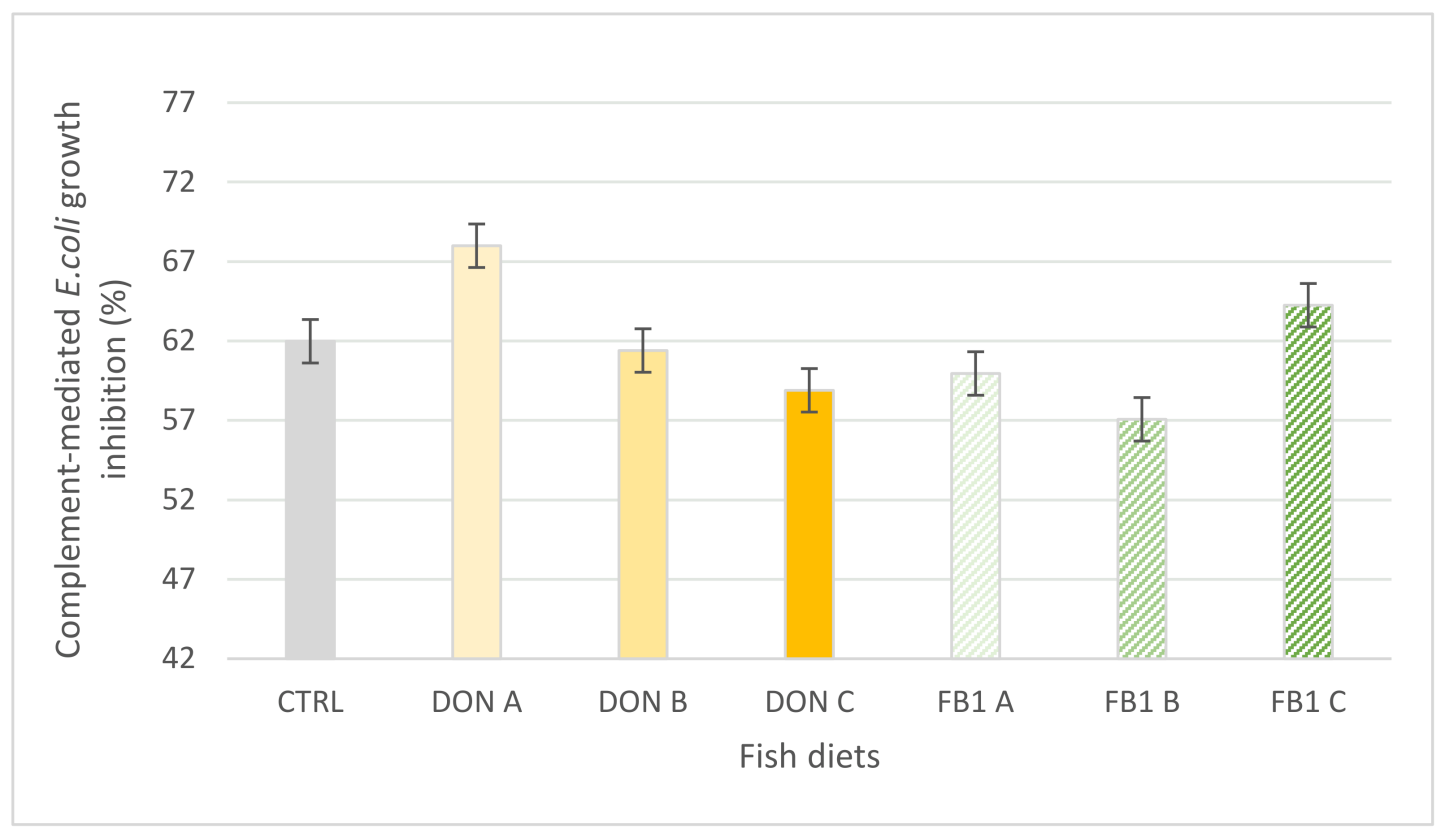
Complement-mediated antibacterial activity of supplement in the serum of gilthead seabream fed with mixtures of different levels of two mycotoxins (*n* = 24). Values are presented as means ± S.E.M. of the percentage of *E.coli* growth inhibition.

Although no statistically significant differences were observed in the percentage of *E. coli* growth inhibition mediated by the complement system in gilthead seabream, in any of the mycotoxin-enriched diets compared to the control diet, a variable trend in complement activity was noted across treatments, as shown in Figure 1 (Kruskal–Wallis, *p* > 0.05). The DON A and FB1 C groups exhibited the highest mean inhibition rates, while the DON C and FB1 B groups demonstrated relatively low levels of activity.

**Figure 2:**
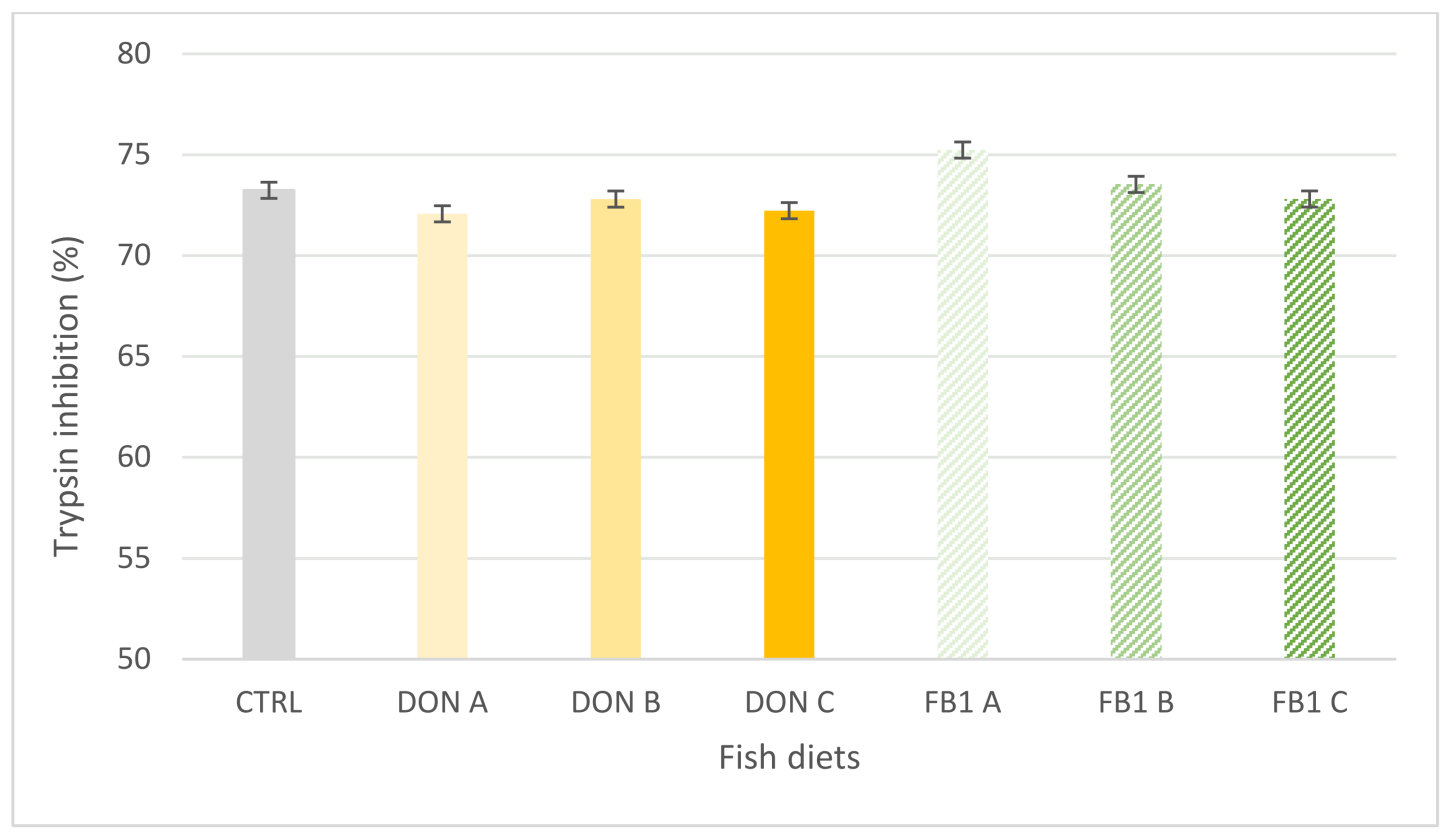
Serum inhibitory activity of trypsin in gilthead seabream fed with mixtures of different levels of two mycotoxins (*n* = 24). Values are presented as means ± S.E.M. of the percentage of trypsin inhibition.

Across all treatment groups, trypsin inhibition remained relatively stable, with no significant difference observed (Kruskal–Wallis, *p* > 0.05), as illustrated in Figure 2.

**Figure 3:**
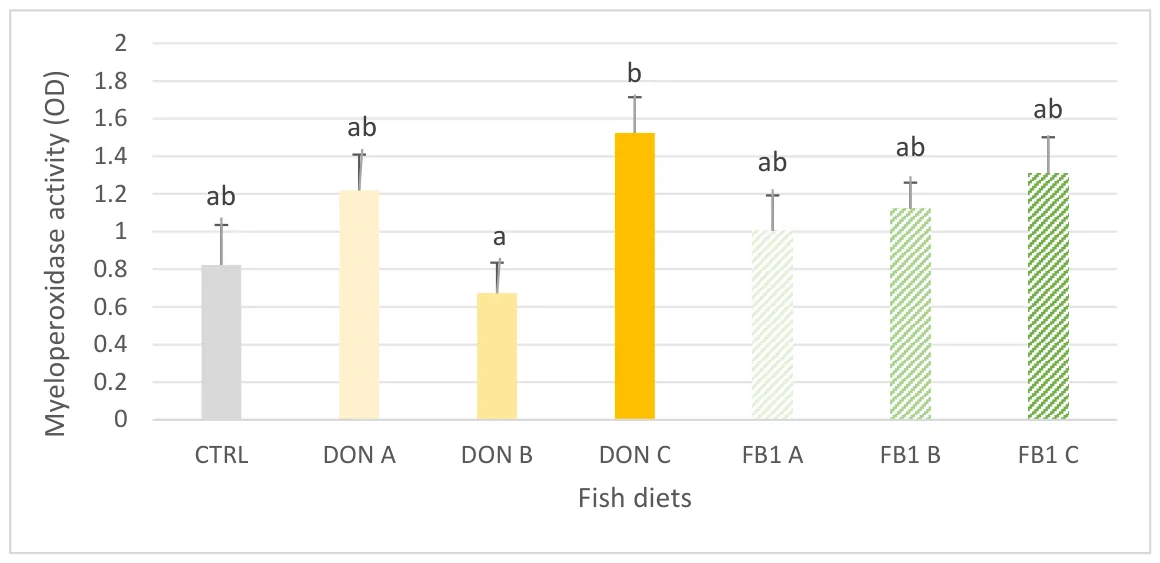
Myeloperoxidase activity (MPO) in serum of gilthead seabream fed mixtures of different levels of two mycotoxins (*n* = 24). Values are presented as means ± S.E.M. of the optical density values. Different letters indicate a statistically significant difference between treatments (Kruskal–Wallis, *p* < 0.001, Games–Howell *t*-test).

A statistically significant increase in myeloperoxidase (MPO) activity was observed in the DON C group compared to the DON B group. Although the FB1 groups did not differ significantly from the CTRL, MPO activity showed an apparent dose-related increase across the FB1 groups (Figure 3).

**Figure 4:**
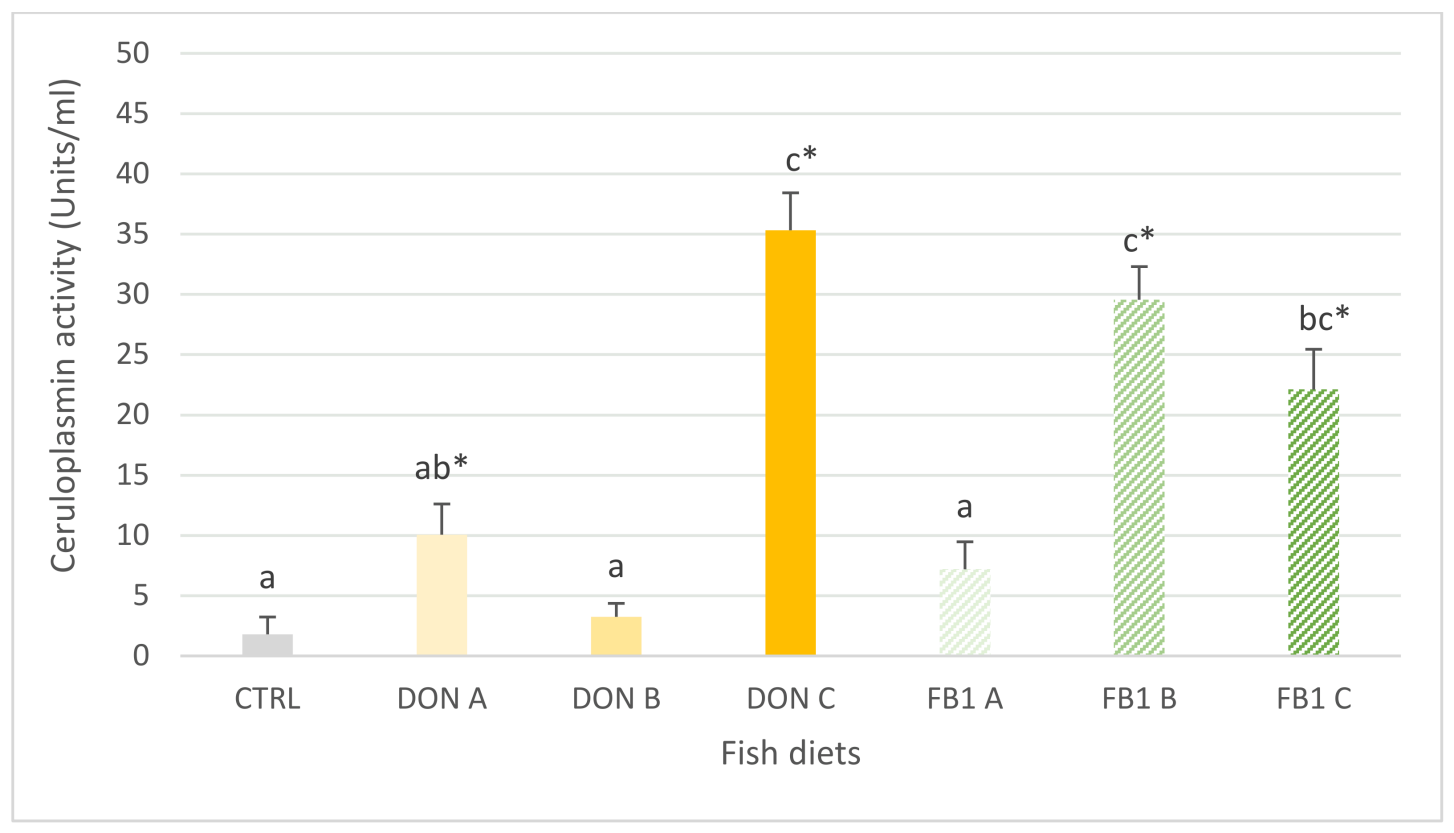
Ceruloplasmin activity in serum of gilthead seabream fed mixtures of different levels of two mycotoxins (*n* = 24). Values are presented as means ± S.E.M. in Units/ML. Different letters indicate a statistically significant difference between treatments (Kruskal–Wallis, *p* < 0.001, Games–Howell *t*-test). Asterisks show significant differences between the shown diets and the control diet without the addition of mycotoxin (simple contrast analysis (*p* < 0.05)).

A significant increase in ceruloplasmin activity in the serum of gilthead seabream was observed in the DON C, FB1 B and FB1 C groups compared to the control group. DON A, DON B and FB1 A did not differ statistically from the CTRL group (Figure 4).

**Figure 5:**
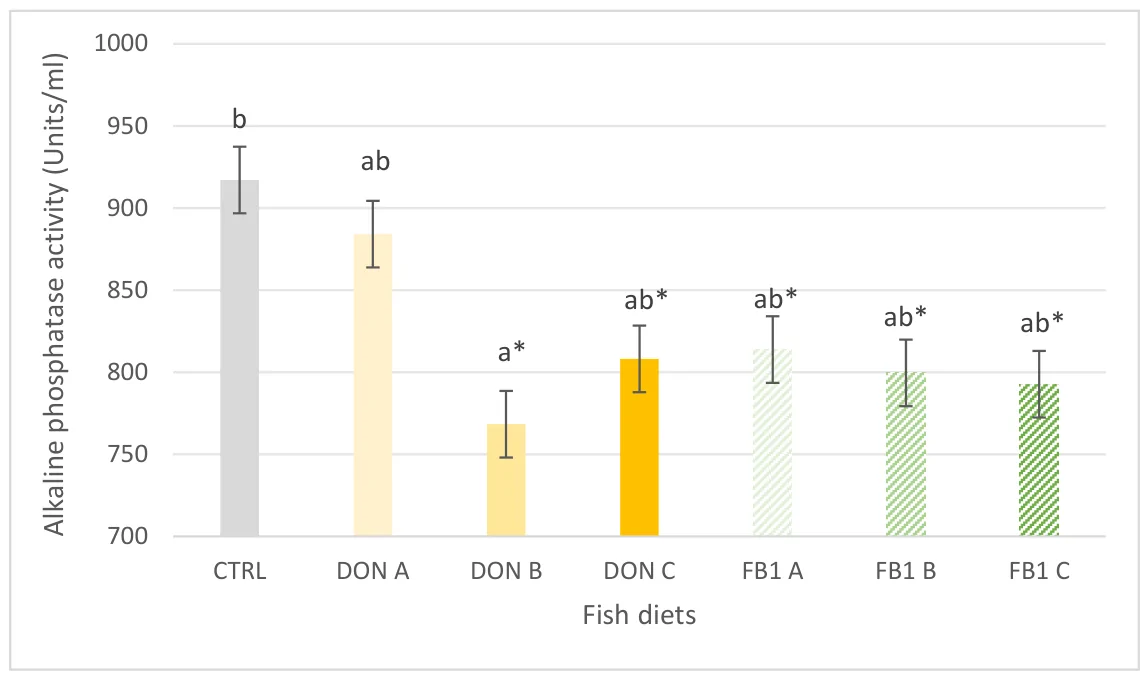
Alkaline phosphatase (ALP) activity in serum of gilthead seabream fed with mixtures of different levels of two mycotoxins (*n* = 24). Values are presented as means ± S.E.M. in Units/ML. Different letters indicate a statistically significant difference between treatments (One-Way ANOVA, *p* = 0.014, Tukey’s *t*-test). Asterisks show significant differences between the shown diets and the control diet without the addition of mycotoxin (simple contrast analysis (*p* < 0.05)).

A significant overall treatment effect was detected for ALP activity (Figure 5). ALP activity was significantly lower in the DON B group than in the CTRL group. Although ALP activity was also reduced in the DON A, DON C, FB1 A, FB1 B, and FB1 C groups compared with the CTRL group, these differences were not statistically significant.

### 3.4. DNA Damage

**Figure 6:**
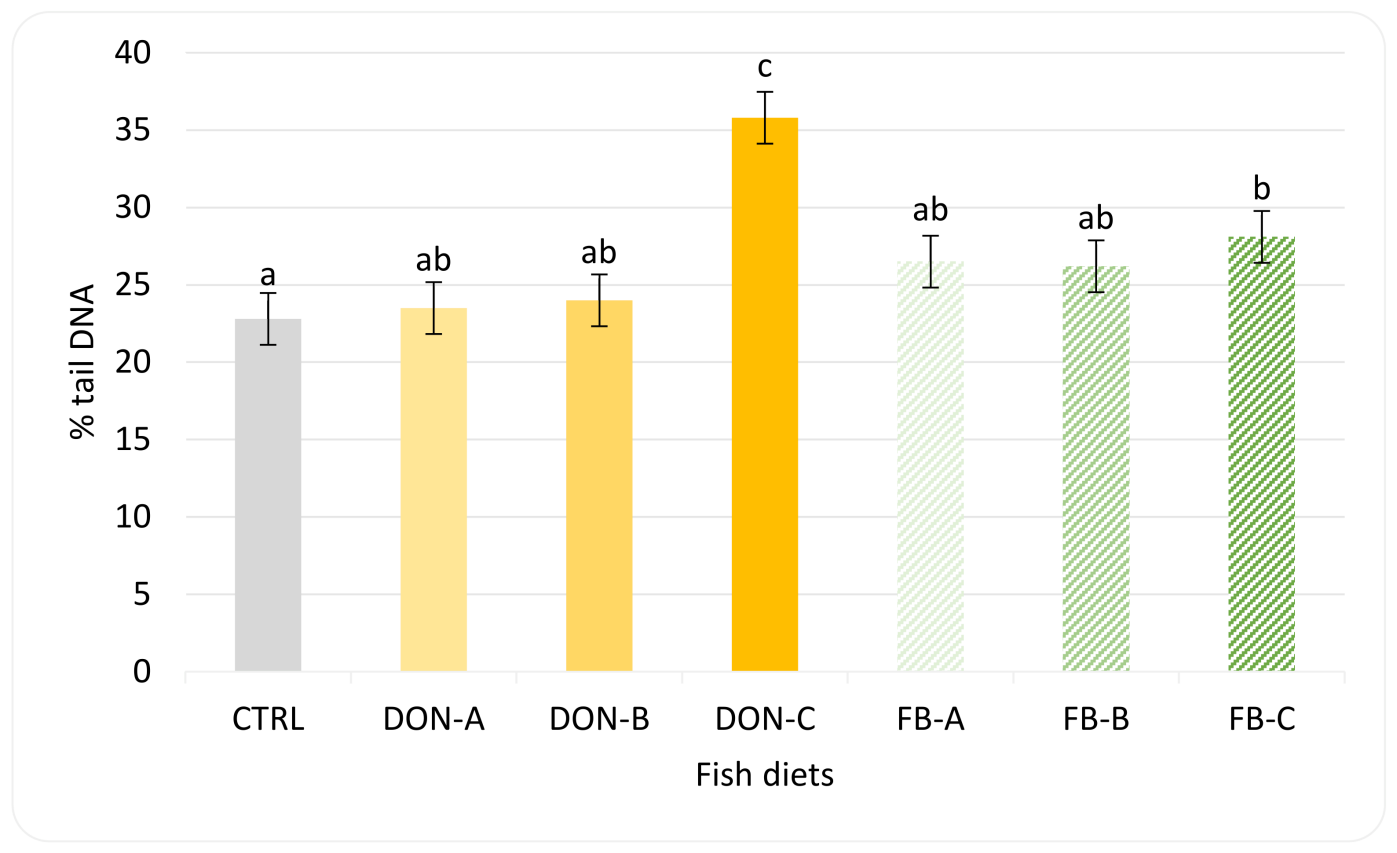
Level of DNA damage according to tail intensity in the hepatocytes of gilthead seabream fed with mixtures of different levels of two mycotoxins. Values are presented as means ± standard deviation, Tail % Intensity, of the triplicate groups of each treatment. In each parameter examined, a symbol with a different letter indicates a statistically significant difference between treatments (*p* < 0.05).

**Figure 7:**
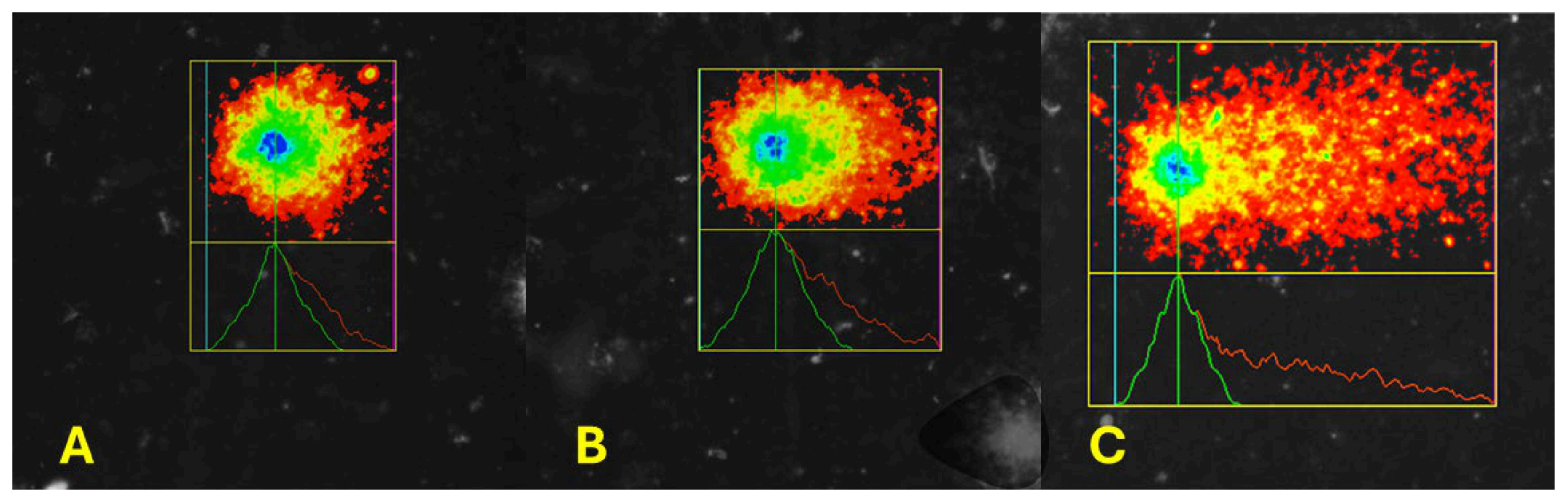
Representative comet assay images (alkaline single-cell gel electrophoresis) illustrating DNA damage in cells from different experimental groups. (**A**) Control group (CTRL), showing a comet with limited DNA migration, with fluorescence intensity in the tail (Tail % Int.) of 24.19% and tail migration of 12.10. (**B**) FB1 B group, displaying increased DNA strand breaks, as indicated by a higher Tail % Int. (32.82%) and tail migration of 19.54. (**C**) DON C group, exhibiting extensive DNA fragmentation with a pronounced comet tail, reflected by a Tail % Int. of 54.94% and tail migration of 50.91. Quantitative image analysis was performed using Comet Assay IV software (version 4.20, Perceptive Instruments, UK). The colored overlays are generated by the Comet Assay IV software: the yellow boxes/lines delineate the regions used for comet analysis, while the green and red/orange profiles represent the fluorescence intensity distribution of the comet head and the migrated DNA toward the tail, respectively.

The genotoxic effects of mycotoxin exposure showed a concentration-related pattern, as indicated by the degree of DNA damage observed across the treatment groups (Figure 6). The comet assay analysis demonstrated an increase in DNA tail migration with increasing dietary mycotoxin concentrations, particularly in DON-exposed fish (Figure 6 and Figure 7). Fish subjected to the highest dose of DON C exhibited the most pronounced DNA damage, with significantly greater DNA migration compared to all other groups, including both the FB1 and control groups. Moderate DNA damage was observed in the intermediate- and low-dose groups (DON A, DON B, FB1 A, FB1 B), with values exceeding those of the control group in some instances but significantly lower than those recorded in DON C. Among all the treatments, DON C elicited the strongest genotoxic response, indicating a particularly potent DNA-damaging effect at elevated concentrations, thereby confirming a clear dose-dependent relationship between dietary mycotoxin exposure and genotoxicity in fish.

### 3.5. Histopathological Examination

**Figure 8:**
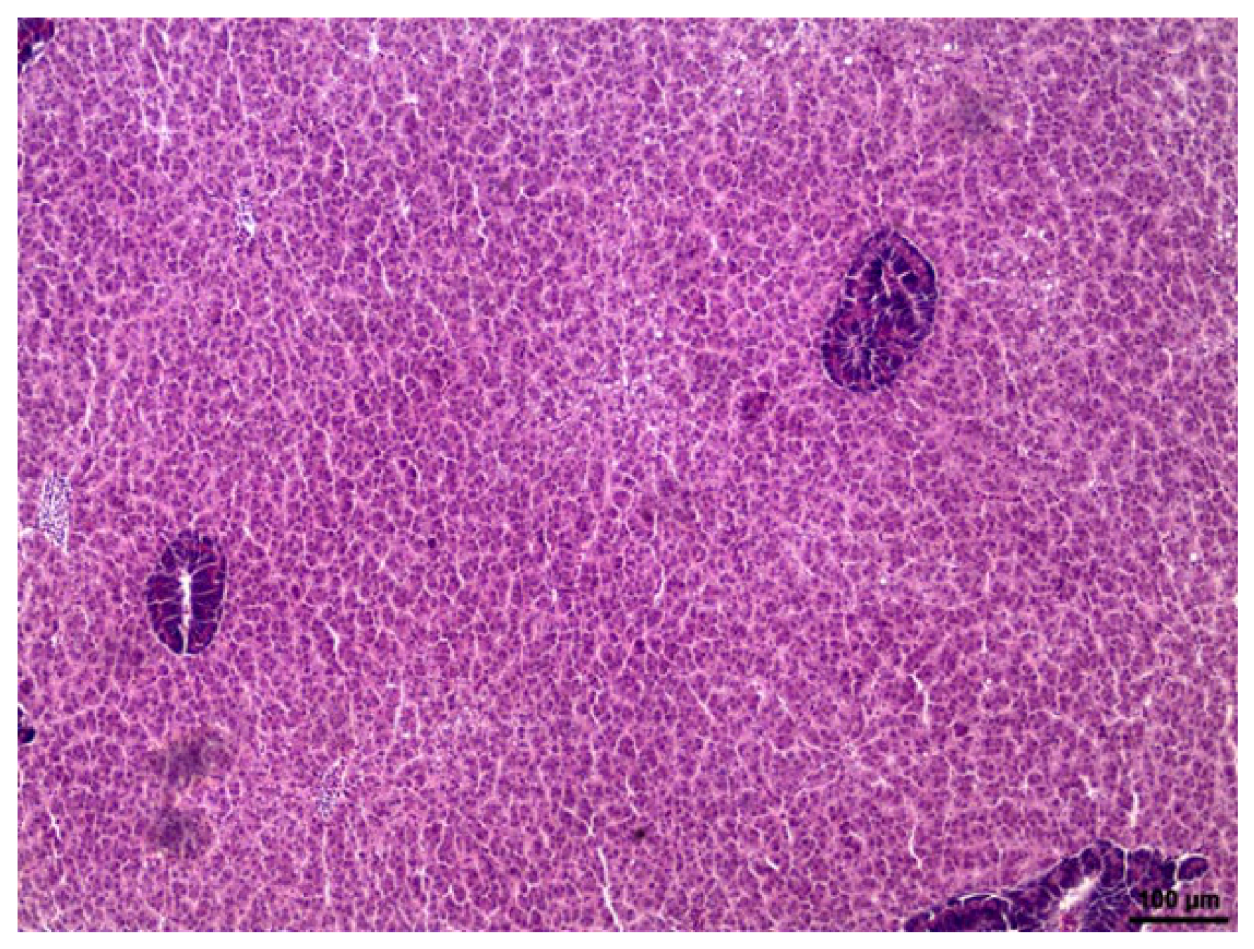
Liver tissue of gilthead seabream (*Sparus aurata*)–HE staining, 100×. CTRL group (fed with a mycotoxin-free diet). Normal liver parenchyma and hepatopancreas.

**Figure 9:**
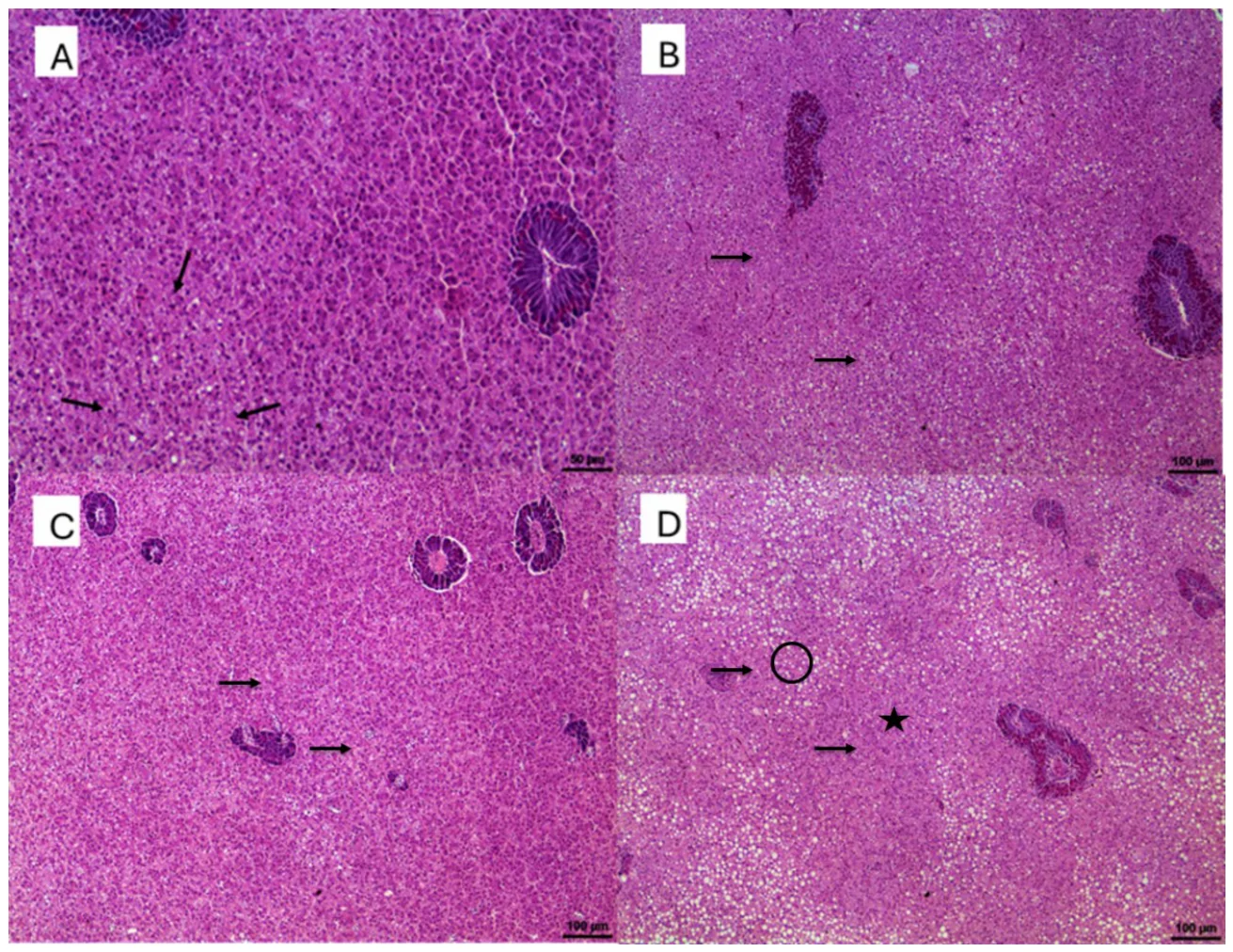
Liver tissue of gilthead seabream (*Sparus aurata*)–HE staining, 100×. (**A**) Group DON A. Mild hydropic and fatty degeneration of hepatocytes in some areas of the liver (arrows). (**B**) Group DON B. Mild and more diffusely distributed hydropic and fatty degeneration of hepatocytes (arrows). (**C**) Group FB1 A. Mild hydropic and fatty degeneration of hepatocytes, affecting specific areas of the hepatic parenchyma (arrows). (**D**) Group FB1 B. Mild to extensive hydropic and fatty degeneration of hepatocytes with wider distribution throughout the liver parenchyma (arrows). Occasional pyknotic nuclei, indicative of early nuclear alterations (circle), along with focal areas showing early necrotic changes (asterisk).

**Figure 10:**
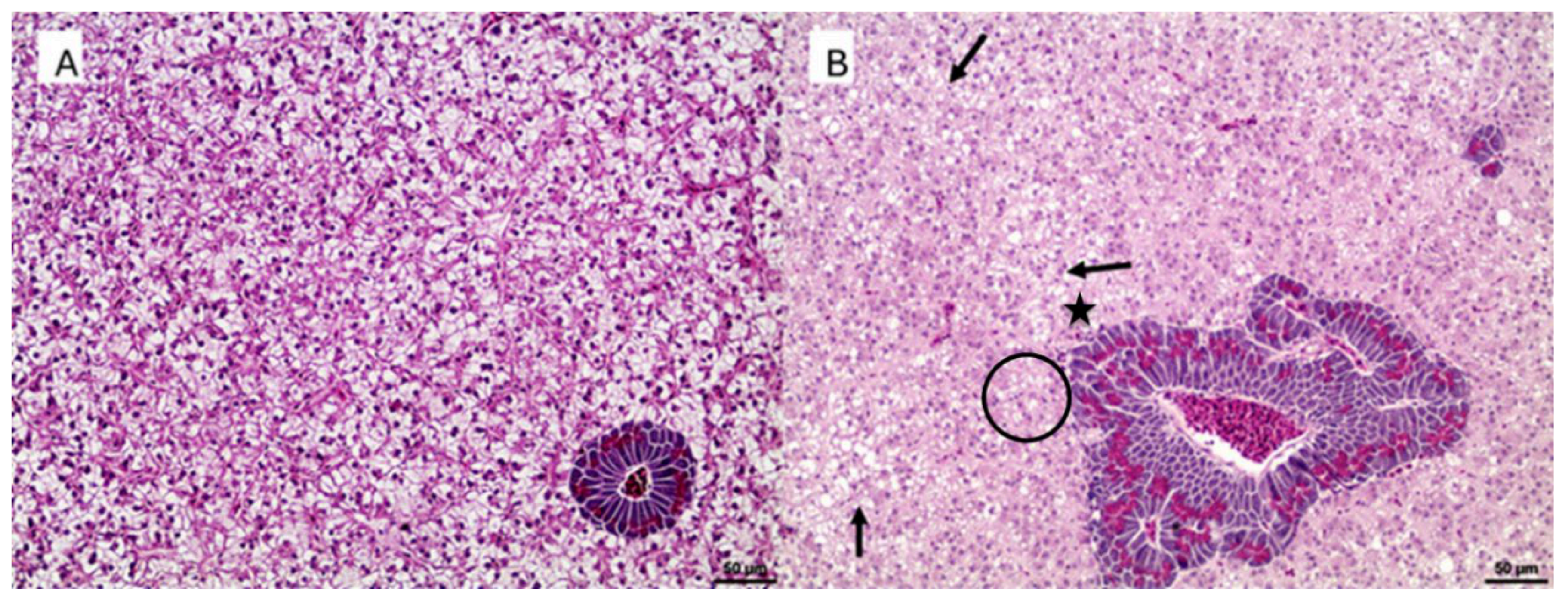
Liver tissue of gilthead seabream (*Sparus aurata*)–HE staining, 50×. (**A**) Group DON C. Diffuse hydropic and fatty degeneration of the liver. (**B**) Group FB1 C. Extensive hydropic and fatty degeneration of hepatocytes, with wider distribution throughout the liver parenchyma (arrows). Occasional pyknotic nuclei, indicative of early nuclear alterations (circle), along with focal areas showing early necrotic changes (asterisk).

Histopathological analysis of the liver tissues demonstrated a distinct contrast between the CTRL and all other groups exposed to both mycotoxins (Figure 8, Figure 9 and Figure 10). Liver sections from the CTRL group exhibited normal hepatic parenchyma and hepatopancreas structures with no observable pathological changes. Conversely, fish from groups fed mycotoxin-contaminated diets, specifically DON and FB1, exhibited varying degrees of hepatic lesions, contingent upon the administered dose. Notably, the low- and intermediate-exposure groups (DON A, DON B, FB1 A, and FB1 B) exhibited mild degenerative alterations in hepatocytes. These changes were characterized by moderate hydropic and fatty degeneration and focal vacuolation of hepatocytes, which was more pronounced in localized liver regions. The severity of the lesions increased significantly in the high-dose group. Liver samples from the DON C group exhibited extensive degenerative pathology, including widespread hydropic and fatty degeneration across multiple hepatic lobules. Similarly, the FB1 C group showed severe and diffuse hepatocellular degeneration, with lesions more uniformly distributed than those in the DON C group. Early signs of hepatocellular necrosis were observed in both high-dose groups, as evidenced by the nuclear pyknosis and cytoplasmic shrinkage. Notably, in several sections, within the FB1 C group, there was a partial loss of the normal hepatic structure, indicating more advanced tissue disintegration. Additionally, mild vascular congestion was noted within the hepatobiliary blood vessels in most of the mycotoxin-exposed groups. However, the pancreatic component of the hepatopancreas remained histologically unremarkable, with no detectable structural alterations in pancreatic acinar cells. These findings underscore the hepatotoxic potential of DON and FB1, particularly at higher dietary concentrations, and they indicate a dose-dependent progression of hepatic injury, ranging from mild cellular degeneration to substantial architectural disruption and early necrosis.

## 4. Discussion

DON and fumonisins (FB1 and FB2) are the most prevalent *Fusarium* mycotoxins in aquaculture feeds, occurring in approximately 58% and 69% of fish feed samples, respectively, and frequently co-occurring (>40%), a pattern that may enhance the risk of combined toxicity [38]. The effects of mycotoxins, particularly DON and FB1, have been extensively investigated in terrestrial livestock [39,40,41,42]. In recent years, the effects of mycotoxins have been studied in aquatic species, such as rainbow trout and zebrafish (*Danio rerio*), but research in this area remains less comprehensive than that of land-based farm animals. Studies focusing on Mediterranean aquaculture species, such as gilthead seabream and European seabass, are significantly less extensive compared to research on other species. Surveys on the natural contamination of aquaculture feeds by mycotoxins are also limited, especially in Europe, and, more specifically, considering gilthead seabream. Limited information makes it challenging to design experimental procedures and determine the optimal experimental administration levels for these species. Few studies have examined FB1 and DON contamination in European aquafeeds and Mediterranean cultured species. Regarding gilthead seabream specifically, elevated levels of FB1 (6400 ppb) and DON (7920 and 5350 ppb) have been found in a gilthead seabream feed sample from Europe [28]. Recent surveys conducted on gilthead seabream and European seabass marketed in Europe have confirmed the natural occurrence of mycotoxins in farmed marine species, underscoring the persistent scarcity of data on contamination patterns within Mediterranean aquaculture systems [29]. Considering the above-mentioned factors, an in vivo feeding trial was conducted under controlled experimental conditions to assess the effects of different dietary concentrations of FB1 and DON on growth performance and health parameters in juvenile gilthead seabream. Juvenile fish (initial mean body weight: 3.40 ± 0.01 g; mean total length: 6.40 ± 0.01 cm) were maintained in a recirculating aquaculture system and exposed through the diet to different concentrations of pure DON or FB1 for seven weeks. The experimental diets were formulated to represent practical aquafeed formulations, while the selected mycotoxin concentrations included levels relevant to current European guidance and lower exposure levels of potential practical relevance.

### 4.1. Growth Performance

Survival rates remained high across all treatments, indicating that the concentrations of applied mycotoxins did not induce acute mortality. However, despite the absence of immediate mortality, significant sublethal effects were observed on performance parameters, particularly at higher levels of contamination, such as reduced feed intake, suppressed somatic growth, lower biomass gain, and deterioration of feed utilization efficiency.

Feed consumption exhibited a dose-dependent reduction in fish exposed to increasing DON concentrations, with the DON C group showing significantly lower total feed consumption and feed consumption per fish compared to all other treatments. This reduction in feed intake was accompanied by consistent decreases in total body weight and length, biomass gain, weight gain per fish, and SGR, suggesting that the reduced growth observed in this group was, most likely, primarily driven by reduced voluntary feed intake, due to decreased appetite [43]. The elevated FCR presently recorded in the DON C group further indicates a deterioration in feed utilization efficiency, reinforcing the negative impact of high DON exposure on overall growth performance. Fish exposed to the highest DON level (DON C) exhibited a reduction in feed consumption per fish of approximately 25%, along with an increase in FCR of ~15–16% and a decrease in SGR of ~20% relative to the CTRL group, further supporting the consistent negative effect of DON on growth performance. Similar anorexigenic effects of DON, leading to reduced growth performance, have recently been reported and associated with impaired intestinal function and compromised nutrient absorption efficiency in largemouth bass (*Micropterus salmoides*) exposed to this mycotoxin [44]. Specifically, juvenile largemouth bass were exposed for 56 days to diets containing 100 or 300 μg DON/kg feed, with the higher concentration resulting in pronounced growth impairment together with hepatointestinal alterations. Comparable findings have been reported in Atlantic salmon, where adult fish (~405 g) were exposed for 15 weeks to dietary DON concentrations ranging from 300 to 3700 ppb, an amount similar to the concentrations applied in the DON B and DON C treatments of the present study; it resulted in a 20% reduction in feed intake, an 18% increase in FCR, and a 31% decrease in SGR [45]. Reduced feed intake associated with DON has also been reported in other fish species, including rainbow trout and Atlantic salmon (*Salmo salar*) [7,46].

A similar, although less pronounced, pattern was observed in the FB1 treatments. While feed intake in the FB1 A and FB1 B groups did not differ significantly from the CTRL, the highest FB1 concentration (FB1 C) resulted in reduced feed consumption per fish and significantly lower growth-related parameters, including total weight, biomass increase, and weight gain. The magnitude of growth impairment in the FB1 C group occurred despite feed intake values that were not as markedly reduced as those observed in DON C, suggesting that FB1 may exert additional metabolic or physiological effects beyond appetite suppression [24]. In the case of gilthead seabream, dietary exposure to FB1 has previously been investigated by Gonçalves et al. [47], who tested moderate to high contamination levels (2000–4000 ppb, considerably higher than those applied in the present study) and reported adverse effects on growth performance. Notably, in the present study, significant growth impairment was observed at substantially lower FB1 concentrations, highlighting the sensitivity of this species to chronic, low-dose FB1 exposure. Recent evidence in gilthead seabream further indicates that FB1 can impair growth performance independently of changes in feed intake, likely reflecting underlying metabolic disturbances rather than a primary anorexigenic effect [48].

When comparing DON and FB1 treatments at comparable performance outcomes, DON provoked a more pronounced decreased appetite, whereas FB1 exposure resulted in growth impairment that may be partially independent of feed intake reduction. This distinction is particularly evident at intermediate contamination levels, where DON B already caused measurable reductions in growth performance, while FB1 B had more moderate effects. These findings suggest distinct modes of action for the two mycotoxins, with DON primarily affecting feeding behavior and FB1 potentially disrupting nutrient utilization and growth-related metabolic pathways [24,44,49].

A mechanism underlying the growth impairment observed in FB1-exposed fish involves the disruption of the sphingolipid metabolism. FB1 is structurally similar to sphingoid bases and inhibits ceramide synthase, resulting primarily in the accumulation of free sphingoid bases, particularly sphinganine and sphingosine, together with alterations in ceramide and complex sphingolipid pools [50,51]. These changes may interfere with membrane organization, cellular signaling, proliferation, apoptosis, and nutrient metabolism, thereby impairing tissue growth even when feed intake is not markedly reduced. Goel et al. [52] provided in vivo evidence of the fumonisin-associated disruption of the sphingolipid metabolism in channel catfish (*Ictalurus punctatus*). Following a 12-week dietary exposure to a *Fusarium* moniliforme culture material containing 0.3–240 mg FB/kg, the free sphinganine-to-sphingosine ratio increased significantly in a tissue-dependent manner, beginning at 10 mg/kg in the kidney, 20 mg/kg in serum, 40 mg/kg in the liver, and 80 mg/kg in muscle. These alterations were primarily attributed to the accumulation of free sphinganine, demonstrating that the sphingolipid-disrupting mode of action of fumonisins is also biologically relevant in fish. Nevertheless, the substantially higher exposure levels and the use of fungal culture material in that study should be considered when extrapolating these findings to the low FB1 concentrations tested in the current study. In addition, FB1-related growth impairment may involve the disruption of endocrine pathways regulating somatic growth. Claudino-Silva et al. [49] reported reduced hepatic expression of growth hormone receptor (GHR) and insulin-like growth factor 1 (IGF-1) in Nile tilapia (*Oreochromis niloticus*) exposed to dietary fumonisin, despite the absence of a corresponding reduction in feed intake. Therefore, the growth effects observed in the present study may reflect a combination of impaired sphingolipid-dependent cellular functions, altered nutrient utilization, and disruption of the GH/IGF axis rather than a solely anorexigenic response. However, since sphingolipid metabolites and GH/IGF-axis markers were not measured in the present study, these mechanisms should be regarded as biologically plausible interpretations requiring direct confirmation in gilthead seabream. The observed reduction in appetite and feed intake in both the DON and FB1 treatment groups occurred at levels lower than the reference concentrations established in European legislation [53]. Specifically, the maximum permitted limit for FB1 in complete and complementary fish feeds is 10,000 ppb, whereas the highest concentration of FB used in our experimental diets that elicited adverse effects was only 40 ppb (FB1 C). This finding, supported by preliminary unpublished evidence of hepatocellular alterations at 10 ppb, is of relevance from a risk-assessment perspective and underscores the need for reconsideration of the current maximum permitted regulatory level for fumonisins in fish feeds to ensure an adequately protective standard for fish health. Regarding DON, although no specific regulatory limit exists for fish feeds, we adhered to the general guidance for complete and complementary feeds for other animals, which sets the limit at 5000 ppb. The maximum concentration of DON used in our diets was exactly this value (DON C), and negative effects were still noted. These findings indicate that both DON and FB1 can have detrimental effects on fish health and performance, even at concentrations far below the current regulatory thresholds. Therefore, reconsideration of the existing mycotoxin legislation may be needed, with greater emphasis on the specific sensitivity of fish species to these contaminants and the combined effects of the presence of more than a single mycotoxin.

Exposure duration is a critical factor in interpreting the observed effects. Although our study encompasses only a segment of the commercial production cycle of gilthead seabream, the consistent decline in growth performance across multiple parameters suggests that even relatively brief exposure to low or moderate mycotoxin concentrations can compromise production efficiency. Recent studies further indicate that measurable effects may develop within a few weeks in juvenile fish. Mello et al. [48] reported tissue-specific biochemical alterations in juvenile gilthead seabream following 28 days of dietary exposure to 150 μg FB1/kg, while Chen et al. [44] observed impaired growth, reduced digestive enzyme activities, and disruption of intestinal integrity in juvenile grass carp exposed to FB1 for 30 days. Production stage may also influence the magnitude of the response. Claudino-Silva et al. [49] found that dietary fumonisin exposure reduced weight gain and worsened feed conversion in Nile tilapia (*Oreochromis niloticus*) fingerlings but did not significantly affect the performance of larger juveniles, although hepatic GHR and IGF-1 expressions were reduced at both stages. These findings suggest that early, rapidly growing stages may exhibit more evident production losses, although direct stage-comparison studies remain limited. The effects of mycotoxin exposure may also change over time. Koletsi et al. reported early DON-induced hepatic lesions in juvenile rainbow trout that were exposed to dietary DON concentrations ranging from 700 to 1600 ppb, partial alleviation during continued restrictive exposure, and renewed aggravation following subsequent ad libitum exposure [54]. In a later study, several hepatic and intestinal alterations associated with DON and other *Fusarium* mycotoxins became more pronounced over an eight-week exposure period, with the most severe changes recorded at the final sampling point [55]. Therefore, longer exposure may intensify some effects, although time-dependent adaptation or non-linear responses may also occur. Evidence regarding reversibility after the withdrawal of contaminated feed remains scarce. In common carp, several oxidative and metabolic alterations induced by DON were largely alleviated after two weeks of feeding an uncontaminated diet, whereas hepatic histopathological damage persisted [56]. Thus, prompt replacement of a contaminated feed batch may limit further exposure and allow the recovery of certain functional parameters, but complete reversal of tissue, hematological, or genotoxic alterations cannot be assumed. Full-cycle and post-withdrawal studies are therefore required to determine whether the effects of chronic low-dose exposure accumulate, persist, or reverse in gilthead seabream. In summary, the results demonstrate that both DON and FB1 negatively affect feed intake, growth performance, and feed efficiency in gilthead seabream in a dose-dependent manner, even in the absence of increased mortality at the tested doses. The observed differences in response patterns between the two mycotoxins further underscore the need for toxin-specific risk assessment and emphasize the importance of considering chronic, low-dose exposure scenarios in aquaculture species.

### 4.2. Immunological and Hematological Factors

Hematocrit values tended to decrease in all groups treated with mycotoxins compared to CTRL, with a significant reduction observed only in FB1 A group. Since hematocrit changes did not follow a consistent concentration-related pattern, the isolated decrease in the FB1 A group may reflect a non-linear or transient physiological response rather than a direct dose-dependent effect. Such hematological alterations may be associated with early inflammatory or stress-related processes induced by mycotoxin exposure [57].

A significant decrease in hemoglobin levels was observed in the DON B group compared to the CTRL, indicating a potential disruption of erythropoiesis or red blood cell integrity at this intermediate DON concentration. In contrast, hemoglobin levels in the DON A and FB1 B groups remained comparable to the CTRL, while the remaining treatments exhibited intermediate values without statistically significant differences. The reduction in hemoglobin in fish exposed to DON suggests that DON may compromise red blood cell functionality, possibly through oxidative damage to hemoglobin or erythrocyte membrane components rather than through a simple reduction in erythrocyte numbers. In line with this interpretation, deoxynivalenol exposure has been shown to markedly increase reactive oxygen species (ROS) levels, leading to lipid peroxidation, protein carbonylation, and DNA damage in exposed organisms, effects that are consistent with oxidative injury to cellular proteins, including hemoglobin [58].

RBC counts were significantly increased by both mycotoxins in a dose-dependent manner. The increase in RBC numbers in DON-exposed fish may represent a compensatory response aimed at maintaining oxygen transport under conditions of oxidative or inflammatory stress. However, when considered together with the observed reduction in hemoglobin levels, this increase in erythrocyte numbers suggests that compensatory mechanisms may not be sufficient to preserve effective oxygen-carrying capacity. Similar patterns have been reported in fish exposed to chemical stressors, where increased RBC counts do not necessarily correspond to improved blood functionality [59].

WBC counts showed marked variability among treatments, with higher mean values recorded in the DON B and DON C groups. Although these differences were not statistically significant, the tendency toward elevated WBC counts may reflect immune cell mobilization in response to inflammatory stimuli.

Increased myeloperoxidase activity was observed in the DON C group compared to DON B group but not to the control group. Myeloperoxidase activity is proportional to the production of peroxide, a potent reactive oxygen species (ROS) that may provoke oxidative stress in the fish cells. The involvement of oxidative stress and free radical generation in the toxic action of mycotoxins has been well documented [60], and an imbalance between ROS and antioxidant defense mechanisms can lead to oxidative damage to DNA, proteins, and lipids—as observed upon exposition to mycotoxins [61]. Such oxidative lesions are commonly observed in fish exposed to a variety of dietary, environmental and chemical stressors, including pesticides, insecticides, heavy metals, changes in dissolved oxygen, pH, temperature, and salinity stressors [62]. However, the levels of the increased MPO might still fall within physiological levels of this parameter.

Ceruloplasmin activity was significantly increased by several mycotoxin contamination levels (DON C, FB1 B, and FB1 C) compared to the CTRL, indicating the presence of an inflammatory response. Elevated ceruloplasmin levels have previously been reported in fish exposed to different pollutants and stressors, including rainbow trout, yellow perch, and common carp [63,64,65], and they are considered part of the acute inflammatory response—as observed in response to infection [66]. The present increased ceruloplasmin activity could reflect such an inflammatory process, although the level of the increase might still be within physiological levels [67].

The DON B group also exhibited significantly reduced alkaline phosphatase (ALP) activity. Because of the ability of ALP to neutralize bacterial lipopolysaccharides, a potent inflammatory molecule, ALP is considered as an anti-inflammatory enzyme with a tissue-protective function [68]. Similar reductions in ALP activity have been reported in Atlantic salmon fed diets containing DON at comparable concentrations (42), supporting the hypothesis that dietary DON contributed to the suppression of this parameter. The ability of DON to simultaneously stimulate certain immune responses linked to ROS production and inflammatory process (e.g., myeloperoxidase, ceruloplasmin) while suppressing parameters linked to anti-inflammatory processes (e.g., ALP) might be interpreted as a mixed immune response involving the polarized response of macrophage M1-/M2-like phenotypes directing the immune response towards an inflammatory response or its resolution [65,66,68]. Such immunological responses may also influence hematological homeostasis, contributing to the heterogeneous blood parameter profiles observed in fish exposed to DON. FB1 treatments showed a general tendency toward reduced ALP activity at all tested doses.

Taken together, the concurrent alterations in immunological parameters (myeloperoxidase, ceruloplasmin, and alkaline phosphatase) and hematological indices (HTC, hemoglobin, RBC, and WBC) suggest a coordinated physiological response involving oxidative stress, inflammation, and immune modulation. These combined immunological and hematological disturbances indicate that mycotoxin exposure may compromise systemic homeostasis, potentially reducing fish health status and resilience to additional stressors or pathogenic challenges; this aspect motivates further research involving challenges to assess the fish’s resistance to diseases.

### 4.3. DNA Damage

A clear dose-dependent genotoxic effect of dietary mycotoxins in gilthead seabream, as evidenced by the comet assay results, was demonstrated. Specifically, fish exposed to the highest dietary concentration of DON (DON C) exhibited the most pronounced DNA damage, with significantly elevated tail intensity values compared to all other treatment and CTRL groups. Intermediate levels of DNA fragmentation were observed in both the mid- and low-dose DON and FB1 groups (DON A, DON B, FB1 A, FB1 B), suggesting that even moderate exposure levels can elicit detectable DNA damage, though to a lesser extent than the DON C treatment. Among all the treatments, the DON C treatment elicited the strongest genotoxic response, highlighting the particularly potent DNA-damaging capacity of DON at elevated concentrations, potentially linked to oxidative stress, as suggested by the increased serum MPO response. These results align with growing evidence that *Fusarium* mycotoxins, including DON and FB1, exert genotoxic effects through oxidative-stress-related mechanisms. For instance, FB1 has been shown to increase the production of ROS, which in turn leads to impaired DNA synthesis and enhanced DNA fragmentation [69,70]. Similarly, DON is known to induce ribotoxic stress and activate signaling pathways such as p38 MAPK, leading to apoptosis and genotoxicity [71]. Moreover, the hypoxic conditions often triggered by trichothecenes such as DON may exacerbate cellular stress and promote senescence via HIF-1α activation [72]. DON-induced DNA damage has been directly demonstrated in hepatic models; Domijan et al. [73] reported a significant increase in DNA strand breaks in HepG2 cells following DON exposure, as assessed by the alkaline comet assay, further supporting the genotoxic potential of DON in liver tissue [73]. Emerging studies also suggest that DON can potentiate the genotoxic effects of other DNA-damaging agents, such as colibactin-producing bacteria, thereby amplifying the risk of mutation and carcinogenesis [74,75]. These findings are further supported by reports of other mycotoxins, such as patulin, which have induced severe oxidative stress-mediated DNA damage in mammalian organs, notably in a dose-dependent manner [76].

Oxidative stress may represent a key secondary mechanism contributing to the observed toxin-induced hepatic alterations. Mycotoxins are known to disrupt the redox balance in fish, leading to oxidative damage of vital biomolecules, resulting in cellular injury and the functional impairment of tissues [77]. In the liver, such oxidative insults may aggravate the effects of direct toxin-induced hepatocellular degeneration, promoting apoptosis, necrosis, and inflammatory responses. It can be inferred that liver dysfunction and hepatotoxicity may exacerbate the overall physiological stress experienced by the fish [36]. As a central organ involved in nutrient metabolism, detoxification, and the maintenance of physiological homeostasis, the liver is often among the first tissues to exhibit signs of toxicosis. Impaired hepatic function may compromise nutrient assimilation and metabolic regulation, compromising fish health, growth performance, and survival. Several studies have shown that excessive ROS production can also activate intracellular signaling pathways related to stress response and inflammation, such as NF-κB and MAPK, further exacerbating tissue damage [78,79]. Moreover, chronic oxidative stress may compromise the regenerative capacity of hepatic tissue and alter the expression of genes involved in detoxification and metabolic regulation. Collectively, these effects can negatively impact vital physiological processes such as nutrient utilization, immune response, and overall growth performance. Thus, oxidative stress should be considered a critical factor mediating the sub-lethal toxic effects of mycotoxins in fish [80,81].

Additionally, beyond direct DNA damage, epigenetic mechanisms may also play a role in mediating the long-term effects of mycotoxin exposure [82]. Alterations in DNA methylation patterns, histone modifications, and non-coding RNA regulation have all been implicated in mycotoxin-induced toxicity, including genotoxic, carcinogenic, and reproductive outcomes across animal models. While the current study focused on primary DNA damage, future research could explore whether similar epigenetic disturbances occur in fish exposed to *Fusarium* toxins. Our data confirm that dietary exposure to DON and FB1 compromises DNA integrity in Sparus aurata and underscore the relevance of oxidative and potentially epigenetic mechanisms in driving this genotoxicity. The observed effects, particularly at high DON concentrations, emphasize the need for regulatory vigilance and further investigation into the long-term implications of mycotoxin contamination in aquafeeds.

### 4.4. Histopathological Examination

Microscopic examinations of liver from fish fed mycotoxin-contaminated diets (DON and FB1 groups) revealed significant pathological alterations compared with the CTRL group. Even at low dietary concentrations, both mycotoxins induced mild degenerative changes in hepatocytes, characterized by moderate hydropic and fatty degeneration, as well as multifocal vacuolation throughout the hepatic parenchyma.

Consistent with these findings, pronounced liver damage has been reported in common carp (*Cyprinus carpio*) exposed to dietary DON concentrations of 352, 619, or 953 ppb for four weeks, followed by a two-week recovery period with uncontaminated feed, with the affected fish exhibiting increased lipid accumulation and severe hyperemia relative to healthy controls [18]. Comparable hepatocellular vacuolation and progressive degenerative changes have also been documented in rainbow trout (*Oncorhynchus mykiss*) exposed to 1166 or 2745 ppb DON for 50 days, as well as to a lower concentration of 367 ppb for 168 days. In some cases, this was accompanied by focal necrosis and hemorrhagic lesions, thereby supporting a dose- and time-dependent pattern of DON-induced hepatotoxicity [83]. Furthermore, rainbow trout receiving DON-contaminated feed displayed marked nuclear alterations, including increased frequencies of pyknotic and pleomorphic nuclei, early necrotic foci, hemorrhage, and inflammatory infiltration, highlighting nuclear condensation and focal necrosis as sensitive early biomarkers of toxin-induced hepatic injury [49]. In this study, juvenile rainbow trout were exposed for eight weeks to naturally contaminated diets containing 700 or 1200 ppb DON or to diets supplemented with pure DON at 800 or 1600 ppb; the first six weeks involved restrictive feeding followed by two weeks of ad libitum feeding. Evidence from other species corroborates this observation. In largemouth bass (*Micropterus salmoides*), dietary exposure to DON at concentrations as low as 300 ppb, comparable to the DON-A treatment applied in the present study, resulted in pronounced hepatocellular vacuolization and nuclear alterations, with a clear dose-dependent increase in hepatic degeneration [28]. This pattern closely mirrors the progressive changes observed herein and reinforces the idea that even low to moderate dietary DON levels, within the range commonly detected in commercial aquafeeds, are sufficient to trigger early hepatocellular injury. Notably, organ-specific susceptibility to DON has also been described. Matejova et al. [84] reported minimal hepatic alterations in rainbow trout following DON exposure, whereas severe pathological changes predominated in renal tissues, suggesting species-dependent and organ-selective toxicodynamic responses. Collectively, these findings highlight the complexity of DON-induced toxicity and emphasize the importance of considering dose, exposure duration, species, and target organ when assessing its hepatotoxic potential.

Scattered necrotic hepatocytes have also been observed in channel catfish following dietary exposure to varying doses of FB1 [85]. Additional evidence of FB1-induced hepatotoxicity in fish is provided by Abu-Hassan et al. [86], who documented pronounced hepatocellular vacuolation, fatty degeneration, sinusoidal congestion, and progressive diffuse hepatic degeneration accompanied by focal necrosis in Nile tilapia following dietary fumonisin exposure. Furthermore, focal hepatocellular necrosis, apoptotic hepatocytes, diffuse hepatocellular degeneration, and mild inflammatory infiltration in common carp fed FB1-contaminated diets confirm the capacity of FB1 to induce degenerative and necrotic liver pathology across fish species [87]. Although it is less extensively investigated compared to DON, existing studies consistently demonstrate that FB1 exposure results in degenerative and necrotic hepatic lesions in fish, indicating a clear hepatotoxic effect. In conclusion, the impact of mycotoxin contamination is strongly related to species-specific susceptibility and exposure conditions, which may influence the severity and nature of hepatic damage, as mycotoxin exposure can induce not only hepatic degeneration but also marked inflammatory responses in the liver [88].

## 5. Conclusions

High concentration of both mycotoxins, DON and FB1, caused significantly lower feed consumption, mean weight, mean length, and total biomass increase compared to other treatments. The results emphasize the importance of assessing whether prolonged exposure, such as that occurring throughout the commercial production period, may intensify these toxic effects. Immunological parameters such as myeloperoxidase and ceruloplasmin activity and hemoglobin and ALP concentrations were also affected in groups with lower concentrations of both toxins. It was demonstrated that mycotoxins may modulate the immune system of fish; notably, DON can stimulate some immune parameters and suppress others. This potential effect of mycotoxins is an area of interest for further research. Moreover, mild to extensive histopathological lesions were detected in liver samples for all groups fed with mycotoxin-contaminated feed. As DON and FB1 toxicity might be species specific, similar experiments are recommended in gilthead seabream and in other Mediterranean farmed fish species, as research is lacking. In addition, toxins do not occur individually in feed, so future studies of combined mycotoxin effects in Mediterranean fish species would also be useful for the aquaculture field.

## Figures and Tables

**Table 1:** Formulation of experimental diets.

Formulation (%)	CTRL	FB1 A	FB1 B	FB1 C	DON A	DON B	DON C
Fish meal	40.00	40.00	40.00	40.00	40.00	40.00	40.00
Poultry byproduct meal	15.00	15.00	15.00	15.00	15.00	15.00	15.00
Soybean Concentrate	10.00	10.00	10.00	10.00	10.00	10.00	10.00
Wheat meal	25.50	25.50	25.50	25.50	25.50	25.50	25.50
Fish oil	9.00	9.00	9.00	9.00	9.00	9.00	9.00
Vitamin and mineral premix	0.50	0.50	0.50	0.50	0.50	0.50	0.50
FB1 (ppb)		5.00	10.00	40.00			
DON (ppb)					300.00	2000.00	5000.00

**Table 2:** Growth parameters. Values are presented as means ± standard deviation of the triplicate groups of each treatment. Different letters in each row indicate a statistically significant difference between dietary treatments (*p* < 0.05), *n* = 3.

Parameters	CTRL	DON A	DON B	DON C	FB1 A	FB1 B	FB1 C
Survival (%)	98.89 ± 1.90	97.78 ± 3.80	100.00	100.00	100.00	98.89 ± 1.90	100.00
Feed consumption (g)	500.44 ± 30.70 ^b^	500.88 ± 26.01 ^b^	452.75 ± 22.97 ^b^	373.10 ± 8.98 ^a^	499.87 ± 14.28 ^b^	463.13 ± 25.94 ^b^	438.49 ± 44.78 ^b^
Feed consumption/fish (g)	16.86 ± 0.76 ^b^	17.11 ± 1.47 ^b^	15.09 ± 0.77 ^b^	12.44 ± 0.30 ^a^	16.66 ± 0.48 ^b^	15.47 ± 1.31 ^b^	14.62 ± 1.49 ^a^
Total length/fish (cm)	11.23 ± 0.35 ^c^	11.10 ± 0.17 ^bc^	10.57 ± 0.15 ^ab^	10.20 ± 0.17 ^a^	10.80 ± 0.17 ^bc^	10.80 ± 0.20 ^bc^	10.83 ± 0.31 ^bc^
Total weight/fish (g)	19.67 ± 1.15 ^c^	17.67 ± 0.58 ^bc^	15.33 ± 0.58 ^ab^	13.67 ± 0.58 ^a^	16.67 ± 0.58 ^b^	17.33 ± 1.53 ^bc^	16.33 ± 2.08 ^ab^
Biomass increase (kg)	475.32 ± 26.77 ^c^	389.36 ± 7.97 ^b^	360.79 ± 23.95 ^ab^	307.10 ± 8.87 ^a^	391.69 ± 26.75 ^b^	408.03 ± 26.21 ^b^	386.39 ± 51.56 ^b^
Weight gain/fish (g)	16.11 ± 1.08 ^c^	14.18 ± 0.80 ^bc^	12.15 ± 0.63 ^ab^	10.23 ± 0.30 ^a^	13.23 ± 0.62 ^b^	13.63 ± 1.45 ^bc^	13.07 ± 1.83 ^b^
FCR	1.05 ± 0.05 ^a^	1.21 ± 0.05 ^bc^	1.24 ± 0.05 ^bc^	1.22 ± 0.03 ^bc^	1.26 ± 0.06 ^c^	1.14 ± 0.05 ^abc^	1.12 ± 0.05 ^ab^
SGR	4.37 ± 0.14 ^c^	4.11 ± 0.12 ^bc^	3.80 ± 0.10 ^ab^	3.47 ± 0.05 ^a^	3.97 ± 0.09 ^bc^	4.02 ± 0.21 ^bc^	3.93 ± 0.19 ^b^

**Table 3:** Hematology parameters. Values are presented as means ± standard deviation of the triplicate groups of each treatment. In each parameter examined, symbol with a different letter indicates a statistically significant difference between treatments (*p* < 0.05).

Parameters	CTRL	DON A	DON B	DON C	FB1 A	FB1 B	FB1 C
HCT	36.13 ± 1.54 ^b^	32.6 ± 1.53 ^ab^	30.71 ± 1.42 ^ab^	32.07 ± 1.30 ^ab^	30.06 ± 1.18 ^a^	32.00 ± 0.86 ^ab^	32.20 ± 1.28 ^ab^
Hb	9.01 ± 1.33 ^b^	9.31 ± 1.95 ^b^	7.64 ± 1.35 ^a^	8.81 ± 1.20 ^ab^	8.34 ± 1.73 ^ab^	9.10 ± 1.48 ^ab^	8.91 ± 1.69 ^ab^
RBC (10^6^ μL^−1^)	2.35 ± 0.14 ^a^	4.66 ± 0.15 ^bc^	4.57 ± 0.1 ^bc^	5.36 ± 0.13 ^c^	2.30 ± 0.10 ^a^	3.66 ± 0.14 ^ab^	4.32 ± 0.14 ^bc^
WBC (10^3^ μL^−1^)	57.56 ± 4.69 ^a^	42.42 ± 2.93 ^a^	70.30 ± 15.67 ^b^	69.58 ± 10.90 ^b^	52.07 ± 6.32 ^a^	51.70 ± 9.59 ^a^	56.80 ± 9.67 ^a^

## Data Availability

Data are unavailable due to privacy considerations.

## Abbreviations

The following abbreviations are used in this manuscript:

ALP	Alkaline phosphatase
ANOVA	Analysis of variance
CTRL	Control
DIAE	Department of Ichthyology and Aquatic Environment
DMSO	Dimethyl sulfoxide
DNA	Deoxyribonucleic acid
DON	Deoxynivalenol
EDTA	Ethylenediaminetetraacetic acid
EMFF	European Maritime and Fisheries Fund
FB1	Fumonisin B1
FB2	Fumonisin B2
FCR	Feed conversion ratio
FELASA	Federation of European Laboratory Animal Science Associations
H&E	Hematoxylin and eosin
HBSS	Hank’s balanced salt solution
HCMR	Hellenic Centre for Marine Research
HCT	Hematocrit
HIF-1α	Hypoxia-inducible factor 1 alpha
MAPK	Mitogen-activated protein kinase
MPO	Myeloperoxidase
MS-222	Tricaine methanesulfonate
NFE	Nitrogen-free extract
NF-κB	Nuclear factor kappa B
RBC	Red blood cell
RNA	Ribonucleic acid
ROS	Reactive oxygen species
SCGE	Single-cell gel electrophoresis
SD	Standard deviation
SEM	Standard error of the mean
SGR	Specific growth rate
SPSS	Statistical Package for the Social Sciences
WBC	White blood cell
WG	Weight gain
